# EHMTI-0057. Anxiety and depression in migraine patients and their relation with impact, severity and frequency of headaches

**DOI:** 10.1186/1129-2377-15-S1-D76

**Published:** 2014-09-18

**Authors:** A Zandifar, F Haghdoost, S Zandifar, M Saadatnia

**Affiliations:** 1 Physiology Research Center and Medical Student Research Center, Isfahan University of Medical Sciences, Isfahan, Iran; 2 Medical Student Research Center, Isfahan University of Medical Sciences, Isfahan, Iran; 3 Physiology Research Center, Isfahan University of Medical Sciences, Isfahan, Iran; 4 Isfahan Neurosciences Research Center, Isfahan University of Medical Sciences, Isfahan, Iran

## Introduction

Several evidences showed anxiety and depression were more prevalent in patients suffer from headache. HIT-6 is a valid and reliable questionnaire for evaluating a wide spectrum of headache burden. Hospital Anxiety and Depression Scale (HADS) is a valid and reliable scale for screening the psychological disorders.

## Aims

The objectives of the present study were to determine the prevalence of anxiety and depression in migraine patients and to assess the relation of frequency, severity and impact of headaches with depression and anxiety.

## Methods

In this cross-sectional study we included migraine patients based on ICHD-2 criteria from four clinics in Isfahan, Iran. HADS, HIT-6 and a symptom questionnaire were fulfilled by the patients. Each patient was asked to determine number of headache days per month (HDPM) and also headache severity by numeric rating scale (NRS). NRS is defined as a scale for severity of pain from zero to 10 that is described by the patient (zero stands for lack of pain and 10 describes the worst pain ever experienced by the patient). Regards to odd questions summation of HADS scores' we determined depression score (patients with sores more than 7 were assumed as depressed) and we calculated anxiety score by summing the even questions of HADS scores (patients with sores more than 7 were assumed as anxious). The student T test was used to compare the mean scores between two groups.

## Results

In this study 126 migraine patients with mean (±SD) age of 32.44±9.94 and 65.1% female gender were included. Of the participants, 100 (79.4%) and 56 (44.4%) had anxiety and depression according to the HADS questionnaire respectively. Patients with depression got significantly higher NRS and HDPM scores (P=0.004 and 0.031 resp.) than those without depression. But HIT-6 total score was not different statistically between them (P=0.125). There were no significant differences between patients with and without anxiety for total HIT-6, NRS and HDPM scores (P=0.275, 0.635 and 0.057 resp.).

## Conclusions

Result of this study showed high prevalence of anxiety (79.4%) and depression (44.4%) in migraine patients. Also we found depressed migraineur express high level of headache severity and frequency in comparison to non-depressed migaineur whereas impact of headache is not different between depressed and non-depressed patients. We found no significant relation between anxiety and frequency or severity.

No conflict of interest.

